# Editorial: The role of epigenetics in neuropsychiatric disorders

**DOI:** 10.3389/fnmol.2022.985023

**Published:** 2022-07-25

**Authors:** Margret Shirinian, Chong Chen, Shusaku Uchida, Nafisa M. Jadavji

**Affiliations:** ^1^Department of Experimental Pathology, Immunology and Microbiology, American University of Beirut, Beirut, Lebanon; ^2^Center for Infectious Disease Research, American University of Beirut, Beirut, Lebanon; ^3^Division of Neuropsychiatry, Department of Neuroscience, Yamaguchi University Graduate School of Medicine, Ube, Japan; ^4^SK Project, Medical Innovation Center, Kyoto University Graduate School of Medicine, Kyoto, Japan; ^5^Department of Biomedical Sciences, College of Graduate Studies, College of Veterinary Medicine, College of Osteopathic Medicine, Midwestern University, Glendale, AZ, United States; ^6^Department of Neuroscience, Carleton University, Ottawa, ON, Canada

**Keywords:** epigenetics, neuropsychiatric, histone deacetylase 2, gene regulation, autism spectrum disorder

Neuropsychiatric diseases are complex diseases that are poorly understood despite collective attempts to explain the neurobiology of these disorders. These changes in behavior may result from but not restricted to defective neuronal or glial development, alterations in neurotransmitters and many other factors which adds to disease complexity. Many studies in recent years focused on identifying the genetic basis of the disease by identifying the loci involved in disease manifestation reflecting changes in brain function. More importantly, recent evidence points to a role for epigenetic regulation in developing neuropsychiatric disorders. Several advanced methods have been developed to understand the link between environmental changes and neuropsychiatric disorders through epigenetic signaling and chromatin remodeling. Since our chromatin is accessible to pharmaceutical treatment, this opens new avenues to alternative treatment modalities of psychiatric diseases. This Research Topic comprises eight papers that highlighted the global epigenetic mechanisms involved in certain psychiatric disorders in addition to some in depth understanding of the effect of selective epigenetic modifiers in mouse models. In addition, some studies focused on the impact of antipsychotic drugs on gene regulation in different cell types while analyzing its pharmaco-epigenomic response. In line within the same therapeutic theme, the effect of blocking histone deacetylase was studied in transgenic mouse models of Alzheimer's disease (AD) and its effect on disease progression.

A review by Lee et al. addressed the use of atypical epigenetic and/or consequent transcriptional alterations as biomarkers of early-stage Schizophrenia. The review highlighted the importance of early stage diagnosis of schizophrenia to prevent disease-associated neural impairments by using well validated biomarkers for schizophrenia. One way of modeling schizophrenia that was suggested is the use of patient-derived iPSC cellular models and organoid technologies. These methods may enhance the assessment of specific cell-subtypes in a wide range of brain cells early in its disease development. Single-cell level analyses have already been used and improved characterization of schizophrenia and this was further elaborated in the review (Skene et al., 2018; Sawada et al., 2020).

A study by Swathy and Banerjee assessed the pharmaco-epigenomic response of antipsychotic drugs using genome-wide microRNA expression. To do so, they have globally profiled the miRNAs influenced by antipsychotic drugs, impacting the first-pass effect on metabolism, using a liver cell line. They have used single or combined treatments of clozapine and haloperidol and assessed differentially regulated miRNA expression. Eight miRNAs were commonly upregulated, and three were commonly downregulated in all conditions. To better understand the biological mechanisms regulated by the antipsychotic drug-induced miRNAs they performed a pairwise pathway analysis and identified a list of miRNAs involved in epigenetic and pharmacokinetic functions.

Histone deacetylase 2 (HDAC2), a member of HADC family, is involved in the epigenetic control of neuronal plasticity, stress response, and cognitive functions (Wood, 2017; Uchida et al., 2018). A study by Nakatsuka et al. investigated if HDAC2 inhibition has neuroprotective and neurorestorative effects in a transgenic mouse model of Alzheimer's disease. The authors first performed HDAC2-specific knockdown in the CA1 region of the hippocampus in APP/PS1 transgenic mouse and then investigated the changes in dendritic morphology and spines. They found that HDAC2 knockdown increased the total length of CA1 basal dendrites, especially in terminal branches, and the density of mushroom-like spines. Furthermore, these structural changes were accompanied by ameliorated impairment in hippocampal CA1 long-term potential and reduced memory deficits in contextual fear conditioning in APP/PS1 transgenic mice. These results suggest the possibility that HDAC2 inhibition may have therapeutic effects for Alzheimer's disease.

A review by Anderson and Taniguchi focused on histone acetylation and histone methylation in a brain region important for drug-related behaviors and also discussed how experimentally altering epigenetic regulators *via* addictive compounds or brain region-specific manipulations demonstrate the importance of epigenetic proteins in the behavioral effects of drugs. They also discussed future directions for the field of epigenetic studies in the behavioral effects of addictive drugs and suggested how to use these insights to develop efficacious treatments.

A study by Du et al. aimed to identify global and site-specific epigenetic changes by antipsychotics and those shared by different classes of antipsychotics. To do this, they performed a comprehensive DNA methylation analysis of human neuroblastoma cells cultured with different antipsychotics (haloperidol and risperidone). This may be the first study to examine the common effect of two distinctive antipsychotics on DNA methylation in human neuroblastoma cells.

A study by Takasu et al. investigated whether histone acetylation regulates kainate-induced gamma oscillations and their important regulator, fast-spiking interneurons, using acute hippocampal slices of AD model mice. They found impairment of gamma oscillation in AD model mice, accompanied with activity of fast spiking interneurons in basal and activated states. HDAC inhibitor SAHA rescued activity of fast spiking interneurons in the activated state in AD mice. The reversal of gamma oscillation deficits by HDAC inhibition appears to be a potential therapeutic target for treating cognitive impairment in AD patients.

A review by Kawatake-Kuno et al. summarized the roles of epigenetic molecules associated with neural plasticity and behavioral regulation in animal models of depression and those that have been suggested to be involved in human major depressive disorder (MDD) patients. They especially focused on sex-related differences in epigenetic signatures in patients with MDD and animal models. They concluded that translational implications for bridging research in human depression and animal models will provide a better understanding of how epigenetic mechanisms contribute to the etiology and pathophysiology of depression.

Autism Spectrum Disorder (ASD) is a complex disease in which the causes remain unknown (Vargason et al., 2020). A study by Rowland et al. reviewed genetic regulation in ASD. The focus of their review was on mutations in the neuronal version of BAF (nBAF). Mutations in this gene (BAF53B) have been shown to lead to the development of ASD and other intellectual disorders. The expression of nBAF is tied to neuronal differentiation and coordination of synaptic genes across brain development. Using neurons from patients with BAF53B when both copies of the gene are deleted led to the disruption of dendritic spine formation. Model systems have shown that the heterozygous loss of Baf53b severely impacts synaptic plasticity and long-term memory. This can be reversed with the reintroduction of Baf53b. Mice that survive the Baf53b deletion display ASD-related behaviors, including social impairments and repetitive behaviors. The review concludes to say mutations in Baf53b and nBAF provide novel avenues to pursue therapeutic advances for treating ASD symptoms in children.

We proposed this Research Topic to highlight the complexity of neuropsychiatric diseases, which are poorly understood despite the collective attempts to explain the neurobiology of these disorders. The studies that were submitted to our Research Topic highlight the many advances in understanding these diseases with potential of therapeutic development. With the advent of new technologies and the ingenuity of clinicians and basic scientists, the future remains hopeful for the treatment of neuropsychiatric diseases.

## Author contributions

All authors contributed to the writing and editing of this Research Topic editorial.

## Funding

MS was supported by grants from the American University of Beirut-Medical Practice Plan (MPP), the Lebanese National Council for Scientific Research (L-CNRS) and the King Hussein Cancer Research Award. NMJ was supported by a grant from the American Heart Association (20AIREA35050015).

## Conflict of interest

The authors declare that the research was conducted in the absence of any commercial or financial relationships that could be construed as a potential conflict of interest.

## Publisher's note

All claims expressed in this article are solely those of the authors and do not necessarily represent those of their affiliated organizations, or those of the publisher, the editors and the reviewers. Any product that may be evaluated in this article, or claim that may be made by its manufacturer, is not guaranteed or endorsed by the publisher.
